# Up-Scaling of Thermomechanically Induced Laves Phase Precipitation in High Performance Ferritic (HiperFer) Stainless Steels

**DOI:** 10.3390/ma14071635

**Published:** 2021-03-26

**Authors:** Jana Pöpperlová, Xiuru Fan, Bernd Kuhn, Ulrich Krupp

**Affiliations:** 1 Steel Institute RWTH Aachen University (IEHK), Intzestr. 1, 52072 Aachen, Germany; Krupp@iehk.rwth-aachen.de; 2 Institute of Energy and Climate Research (IEK), Microstructure and Properties of Materials (IEK-2), Research Centre Jülich, 52425 Jülich, Germany; x.fan@fz-juelich.de (X.F.); b.kuhn@fz-juelich.de (B.K.); 3 Central Iron & Steel Research Institute (CISRI) Group, Material Digital R&D Centre, Beijing 100081, China

**Keywords:** high chromium ferritic steel, intermetallic phase, Laves phase, thermomechanical treatment, precipitation, forging parameter up-scaling

## Abstract

Fully ferritic stainless steels, strengthened by Laves phase precipitates, were developed for high-temperature application in the next generation of ultra-super-critical thermal power plants. Based on the rapid occurrence of thermomechanically induced precipitation in strengthening Laves phase particles, a novel thermomechanical process route for this class of steels was developed. A controlled precipitation of particles, in a desired morphology and quantity, and an optimization of the corresponding forging parameters was achieved on a laboratory scale. This article outlines the very first up-scaling experiment with these optimized forging parameters from the laboratory scale to the industrial scale. The precipitation behavior was analyzed, utilizing digital particle analysis of scanning electron microscopy (SEM) images, to estimate and compare the phase fraction of the precipitated Laves phase, as well as the particle size and distribution. Due to the up-scaling in the forging process, the behavior of the precipitation changed and the precipitation strengthening effect was decreased, in comparison with the laboratory scale.

## 1. Introduction

The fully ferritic, high-chromium stainless steels were designed in recent decades in reaction to the demanded reduction in CO_2_ emissions, by improving the efficiency of future power plants. The high application temperature of 650 °C (300 bar) in the next generation of power plants requires improved material properties such as steam oxidation resistance and creep strength. The novel High Performance Ferritic (HiperFer) steel class is based on alloying elements such as tungsten, niobium and silicon, to support the precipitation of the strengthening intermetallic Laves phase (Fe,Cr,Si)_2_(W,Nb). The favorable combination of a solid solution and Laves phase precipitation strengthening of the HiperFer steels leads to a steam oxidation resistance that is superior to the Advanced Ferritic Martensitic (AFM) steels currently used in power plants [1,2] and to an advantageous thermomechanical fatigue resistance [1,3]. A cyclic deformation at a high temperature induces further precipitation of the Laves phase. Therefore, the HiperFer steels provide an excellent fatigue life, based on a stable microstructure even under demanding operation conditions [1,4,5]. Furthermore, HiperFer steel features a fully ferritic microstructure without undergoing martensitic transformation in general, as well as in the welding process [5,6], i.e., it is intrinsically free from type IV heat-affected zone cracking.

**Figure 1:**
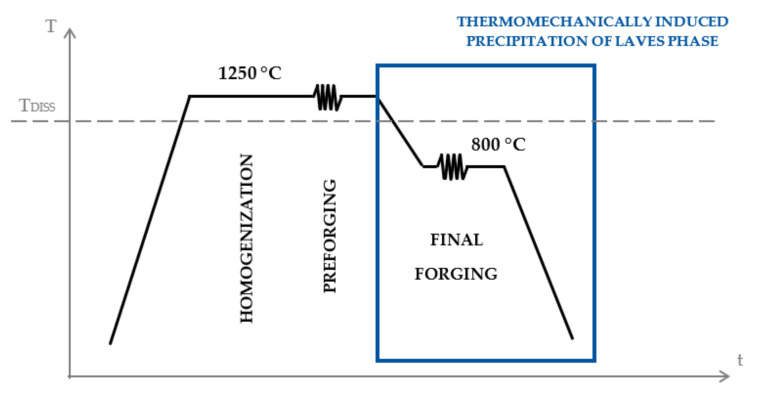
Newly developed thermomechanical processing route of the HiperFer stainless steels with indicated Laves phase dissolution temperature T_DISS_.

The present work is part of a project that aimed to further optimize the chemical composition [7] and development of a new thermomechanical processing route [8,9] of HiperFer stainless steels. This article focusses on the thermomechanically induced precipitation (during final forging) of the Laves phase as part of the complete thermomechanical processing chain [8], as schematically shown in Figure 1. A controlled thermomechanically induced precipitation of Laves phase particles, in a desired morphology and quantity, as well as an optimization of corresponding forging parameters, was achieved on a laboratory scale [8,9]. The present work specifically deals with the very first up-scaling experiments of the optimized forging parameters, from a successful laboratory scale to an industrial scale, to enable a proper standard characterization of the achieved mechanical properties.

## 2. Materials and Methods

### 2.1. Materials

The chemical composition of the studied HiperFer steel is given in Table 1. The steel chemistry of 1.0 wt. % Nb and 2.6 wt. % W was designed [7] to support the precipitation process and stabilize the Laves phase particles. The high-purity model alloy was manufactured at the Steel Institute IEHK by 2 kHz vacuum induction melting. After a homogenization treatment, the 140 × 140 × 535 mm^3^ ingot was hot-forged in three steps into a bar of a 92 × 92 mm^2^ cross-section, utilizing a 400 t automatic hydraulic press. Specimens were taken out of the bar for laboratory-scale forging experiments. Additionally, segments of this bar were used for forging on an industrial scale.

### 2.2. Thermomechanical Treatment

The laboratory-scale forging experiments were performed at the IEHK utilizing a Thermomechanical Treatment Simulator TTS 820 (TA Instruments, Hüllhorst, Germany). This equipment allows an experimental simulation of compressive forging processes, based on a 15 × 15 × 65 mm^3^ sample, from which secondary small-sized tensile and Charpy specimens can be obtained after processing. The performed treatment (chosen regarding the laboratory experiments described in [8]) consisted of dissolution annealing at 1200 °C for 40 min, to dissolve all Laves phase particles formed during the slow cooling of the casted block, followed by a single step forging at the deformation temperature of 800 °C. The TTS sample was deformed, with a deformation grade of φ = 0.2 or φ = 0.5, applying a constant deformation rate of 10 s^−1^. Subsequently, a holding for 60 s at the deformation temperature of 800 °C was carried out. The sample was finally quenched at a quenching rate of 150 °C·s^−1^ by argon. The laboratory-scale forging experiment in the TTS facility was accomplished using an argon-inert gas atmosphere.

The industrial-scale compressive forging experiments of the bar segments (92 × 92 × 250 mm^3^) were performed at the Institute for Metal Forming (IFM), at TU Bergakademie Freiberg, utilizing an oil-hydraulic forming press (Wepuko Pahnke, Metzingen, Germany), with a maximum compression force of 10 MN. The bar segments were dissolution annealed at 1200 °C for 60 min (a prolonged annealing time because of the increased size of the bar segments) and subsequently unilaterally forged at 800 °C in two steps with a total deformation grade of φ = 0.5. To finalize the forging process, the bar segments (final dimensions measuring 55 × 100 × 390 mm^3^) were quenched in water.

For further microstructural and mechanical investigations, samples were taken from the middle part on both the laboratory- and industrial-scale. These areas provided a homogeneous deformation distribution.

### 2.3. Microstructural Investigations

The achieved microstructure was investigated by using a Zeiss Σigma SEM equipped with an in-lens detector for high-resolution imaging, and an Oxford Instruments X-Max 50 EDX-detector for chemical analyses. Backscattered electrons (BSE) imaging was applied for the investigation and qualitative analysis of the precipitation state. All samples were examined in a polished state, due to their soft ferritic structure, in order to ensure an appropriate reproducibility. Based on the high content of high atomic number elements in the precipitated Laves phase, the polished state was sufficient to ensure an adequate contrast between the matrix and the analyzed particles. Secondary electrons (SE) imaging was selected for the investigation of the fracture surfaces that resulted from the Charpy impact tests.

The high-resolution SEM images with identical contrast conditions were used for a quantitative particle analysis by applying the image analysis program AnalysisPro^®^. This software applies the equivalent circle diameter (ECD) method to evaluate the size of the precipitates. All particle analysis data refer to the precipitates in the grain interiors, as the particles precipitated at the grain boundaries are mostly located too closely together, and therefore differentiation between individual particles is difficult or even impossible. Due to the limited resolution of the SEM images, all precipitates below 10 nm were excluded from the evaluation. Clearly identified inclusions, such as niobium oxides, were also considered as irrelevant for the particle analysis. The phase fraction of the Laves phase was evaluated as a percentage area fraction, also referring to the grain interior, without consideration of the particle-free zones (PFZ) along high-angle grain boundaries. This method is in accordance with the particle analysis procedure reported in [10,11].

### 2.4. Mechanical Testing

The tensile tests were performed at an ambient temperature, according to the standard [12], utilizing an electromechanical Zwick Roell Z100 universal testing machine (Zwick Roell, Ulm, Germany). Due to the differences in dimensions of the forged specimens on both the laboratory and industrial scale, the small-sized specimen from the laboratory forging tests (B3 × 15) and the standard sized specimens (B6 × 30) from the industrial forging trial were tested.

In the case of the industrial forging trials, the Charpy V-notch impact tests were performed. The Charpy impact tests were carried out in accordance with the standard [13], utilizing a Düsseldorfer Maschinenbau impact testing machine (Düsseldorfer Maschinenbau, Düsseldorf, Germany) with a maximum impact energy of 300 J and standardized specimens of 10 × 10 × 55 mm^3^ in size. Regarding the grain size of these full-ferritic HiperFer steels, and the size of possible secondary Charpy test samples manufactured from the specimens forged on the laboratory scale, convincing results were not expected. Therefore, only the standard-size Charpy tests of the industrial forged material were provided.

### 2.5. Creep Testing

From the laboratory-scale forged bars (15 × 15 × 65 mm^3^), miniature cylindrical compression creep specimens (d: 3 mm, h: 3.5 mm) were cut by electrical discharge machining (EDM). Full-size, uniaxial creep specimens (gauge diameter: 6.4 mm, gauge length: 30 mm) were machined from the industrial scale forged bar segments (55 × 100 × 390 mm^3^). Stepped stress-compression creep experiments (details on experimental technique are given in [14]) were accomplished applying an Instron 8862 testing machine. The decrease in effective creep stress during the experiment by specimen strain was neglected but limited by the restriction of the maximum total strain to 2%. Creep deformation of HiperFer steel, in the primary and secondary creep regime, is controlled by the growth of the Laves phase precipitates [15]. During long-term service, particle-free zones (PFZs) evolve along high-angle grain boundaries [16], while the grain boundaries remain well occupied by Laves phase precipitates, which effectively inhibit grain boundary slide. Accumulation of plastic deformation within the PFZs governs damage and failure in the tertiary creep regime, which is avoided in miniature specimen testing by the limitation of maximum strain. For this reason, issues in grain size, i.e., specimen size, do not play a significant role in this case. The creep experiments at uniaxial, full-size specimens were carried out in single-specimen, constant-load, lever-arm type creep machines, with continuous elongation measurement at the gauge portions of the specimens. To control the testing temperature to an accuracy of +/−1 °C, type S thermocouples were attached to the specimens in both of the experimental set-ups.

## 3. Results and Discussion

### 3.1. Microstructure

**Figure 2:**
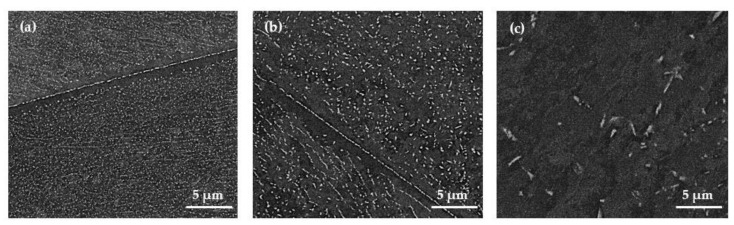
Representative SEM-BSE micrographs of thermomechanically induced Laves phase precipitation in the model alloy Fe17Cr2.6W1Nb created by deformation temperature at 800 °C (**a**) laboratory-scale forging—deformation grade of φ = 0.5; (**b**) laboratory-scale forging—deformation grade of φ = 0.2 and (**c**) industrial-scale two-step forging—final deformation grade of φ = 0.5.

The thermomechanical treatment significantly accelerates the precipitation process by increasing the number of nucleation sites due to the creation of dislocations. As a result, the distances in the diffusing atoms to the nucleus are shortened. Accordingly, fine homogeneously distributed Laves phase particles are precipitated. Based on the forging experiments on the laboratory scale, i.e., deformation at 800 °C, by a deformation grade of φ = 0.5, and subsequent holding for 60 s after deformation, leads to the desired fine homogeneous distribution of precipitates (Figure 2a). Since the industrial-scale experiments were performed in a two-step forging process with a final deformation grade of φ = 0.5, the microstructure, after forging by a deformation grade of φ = 0.2 on the laboratory scale, is additionally displayed (Figure 2b). The impact of an increased deformation grade is clearly visible: a higher deformation yields a higher number of finer particles. Laboratory scale deformation by φ = 0.5 results in a mean ECD of 44 nm, while deformation by φ = 0.2 yields a mean particle diameter of 79 nm. Nevertheless, deformation by φ = 0.2 already leads to precipitation of fine Laves phase particles. The detailed impact of the forging parameters (temperature, deformation grade, and holding time after the deformation) on the precipitation behavior is reported in [8].

**Figure 3:**
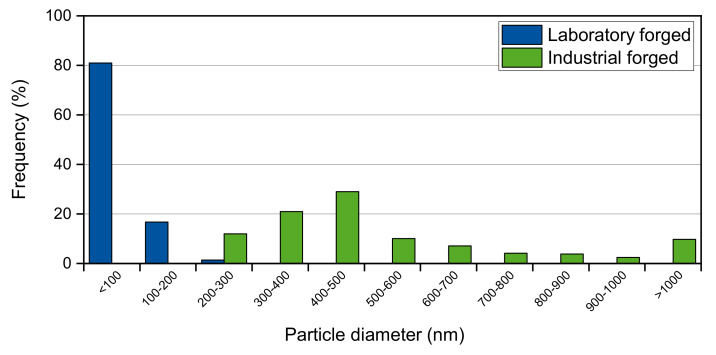
Particle size distribution of intergranular Laves phase precipitates after laboratory and industrial-scale forging experiments at 800 °C and with a deformation grade of φ = 0.5.

The industrial-scale forged microstructure (Figure 2c) exhibits significantly coarser precipitates. Partially rod-shaped Laves phase particles can be observed in the grain interiors. A pronounced deformation microstructure with shear bands, like in the laboratory-scale forged specimens, was not encountered. The grain boundaries were found to be almost completely occupied by comparatively coarse precipitates and surrounded by wide particle-free zones (PFZ). The mean width amounts were 0.6 µm on the laboratory scale and 5.9 µm on the industrial scale. Due to the more visibly inhomogeneous microstructure, higher measurement deviations were provided on the industrial scale. A mean particle diameter of 482 nm and a Laves phase fraction of 1.9% were evaluated for the industrial scale forging. According to the representative microstructure images presented in Figure 2, the laboratory-forged specimens with a deformation grade of φ = 0.5 provide considerably finer precipitates (mean particle diameter of 44 nm) in a significantly higher amount (3.1% phase fraction). The substantial difference can also be observed in Figure 3, which shows a comparison of the particle size distributions after forging experiments on both a laboratory and industrial scale. In general, the laboratory forging process created precipitates below 300 nm in diameter. Furthermore, over 80% of all the analyzed Laves phase particles provide a diameter smaller than 100 nm. In comparison, the industrial forging trials contain evidently coarser particles with a minimum particle diameter above 200 nm.

Due to the larger cross-section of the industrial trial bar, the dissolution annealing was extended by 20 min and the deformation grade of φ = 0.5 was accomplished in two individual steps (while deformation of φ = 0.5 was applied in a single step in the laboratory scale experiments). The first deformation step of the industrial forging trial creates dislocations, and therefore nucleation sites, for Laves phase precipitation. The number of these nucleation sites is lower in comparison to the laboratory one-step forging process. The nuclei grow rapidly because of the high supply of available solute atoms from the supersaturated matrix. The second deformation step introduces additional dislocations, which triggers the formation of further Laves phase particles fractions. Besides the dislocations created by the second deformation, the particles nucleated during/after the first deformation serve as additional nucleation sites. As a result, the first particle fraction grows rapidly during the second deformation step. Furthermore, in comparison to one-step laboratory process the two-step industrial scale forging process resulted in prolonged tempering, which additionally contributes to the particle growth.

### 3.2. Mechanical Properties

**Figure 4:**
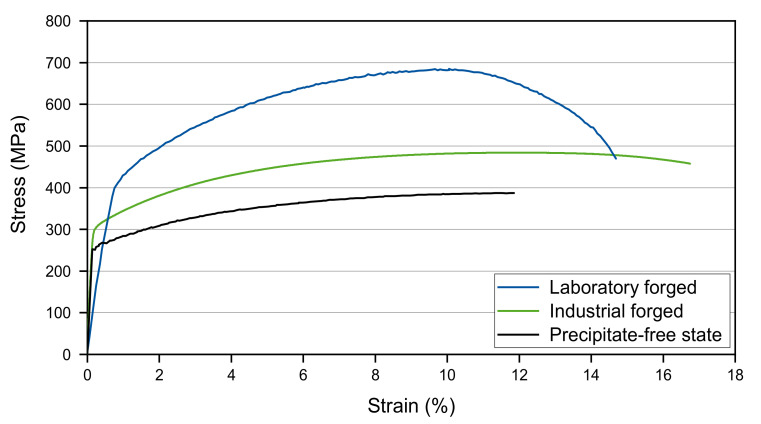
Tensile testing results of the Fe17Cr2.6W1Nb model alloy after the laboratory and the industrial forging experiments in comparison to the solution treated (i.e., undeformed, precipitate-free) state.

The mechanical properties at an ambient temperature were determined for both forging conditions in order to assess the strengthening effect of the microstructures obtained. Representative technical stress-strain curves obtained for both of the forging process routes are compared to the solution-treated state (i.e., particle-free, without deformation) in Figure 4. Significant strengthening by combined work-hardening and Laves phase precipitation was obtained in both the laboratory as well as in the industrial-scale forging. The tensile test results show that the stress–strain curves are characterized by a continuous transition from the elastic into the plastic regime. The model alloy exhibits a yield strength of 412 MPa and an ultimate tensile strength of 683 MPa on the laboratory scale, and reaches a uniform elongation of 9.0% and a total elongation of 14.1%. A microstructure with coarser and fewer precipitates was produced by industrial-scale forging. Correspondingly, a lower ultimate tensile strength of 490 MPa and yield strength of 329 MPa, with a uniform elongation of 10.7% and a total elongation of 17.2%, were achieved. The deviation in Young’s modulus is caused by the miniature specimen size taken from the laboratory forging specimens. Due to the small dimensions in combination with a large grain size, a strong grain orientation dependency of the Young’s modulus, due to elastic anisotropy, can be observed.

**Figure 5:**
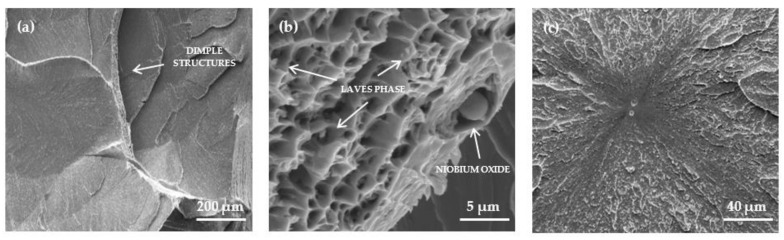
Representative SEM-SE micrographs of fracture surfaces from Charpy impact specimens of the model alloy Fe17Cr2.6W1Nb: (**a**) fracture surface with large cleavage planes; (**b**) dimple structures (indicating areas of ductile rupture) on the steps between the cleavage planes and (**c**) origin of fracture.

In addition to the tensile tests, Charpy impact experiments were performed on industrial-scale forged specimens to investigate the toughness of the material. The industrial forged model alloy provides a very low impact energy of 9 J on average and can be considered as very brittle. Representative fracture surfaces of the notched Charpy impact specimens tested at an ambient temperature are shown in Figure 5. The surface exhibits typical features of transgranular cleavage fractures with large cleavage facets (Figure 5a). Dimple structures are located on the steps between the cleavage planes indicating areas of local ductile rupture. Inside these structures, coarse spherical and small angular particles were found (Figure 5b). By means of EDX analysis, these particles were classified as large niobium oxides and Laves phase precipitates. By tracing fracture propagation, the initiation point of cracking could be identified (Figure 5c). In all cases, the fracture originated from pit-like structures, with coarse spherical particles, presumably niobium oxides, located in the center.

The correlations between chemical composition, processing, heat treatment, and the resulting microstructure and impact toughness of ferritic stainless steels are complex. If proper processing and heat treatment are applied, technically viable impact strength is obtainable [17,18,19]. Large grain size, for example, typically results in a decrease in impact toughness [19], and through water quenching a desired DBTT downshift is accessible [18]. According to the literature [17,18,19], coarse precipitates (in our case Laves phase) at the grain boundaries and within the grain interiors, in combination with large grain size, caused by the chosen two-step process route, by trend result in the encountered low impact toughness values. Additionally, large niobium oxides were identified as the origin of cracking during impact testing.

The toughness can be optimized by an adaptation of the forging parameters on an industrial scale to achieve finer precipitation of the strengthening Laves phase and decreased grain size. These can be achieved by a reduction in the cross-section, accelerated cooling, and further process optimization. Further improvements may be accessible through reduced oxygen content and consequently, avoidance of niobium oxide formation. Considering the complete manufacturing process (see Figure 1), finer grain size can be obtained within the first step of forging (pre-forging), which is performed above the dissolution temperature of the Laves phase at about 1250 °C. Therefore, dynamic recrystallization, as well as the dissolution of all present Laves phase particles, is expected. As a result, the finer grain structure can be deformed within the final forging step.

The optimization of grain size implies a modification in the width of the PFZs. Wide PFZs generally provide a negative impact in the mechanical properties and durability of the material [20,21]. The absence of particles causes these zones to be softer than the surrounding precipitation-hardened matrix. Plastic deformation is concentrated in these zones close to the grain boundaries, increasing the risk of grain boundary fracture [21]. The same applies for creep fracture of lower alloyed Laves-phase-strengthened ferritic steels [11,22]. Consequently, the PFZs may negatively affect the material performance. However, a positive effect on the ductility of wider PFZs was observed in strongly precipitation-hardened alloys [20,23,24]. The investigated high Nb-alloyed HiperFer variant reveals a pronounced hardening by Laves phase precipitates. Therefore, an optimal width of the PFZs must be determined, considering the phenomenon of the positive effect of wider PFZs on the ductility of the material mentioned above.

### 3.3. Creep Properties

**Figure 6:**
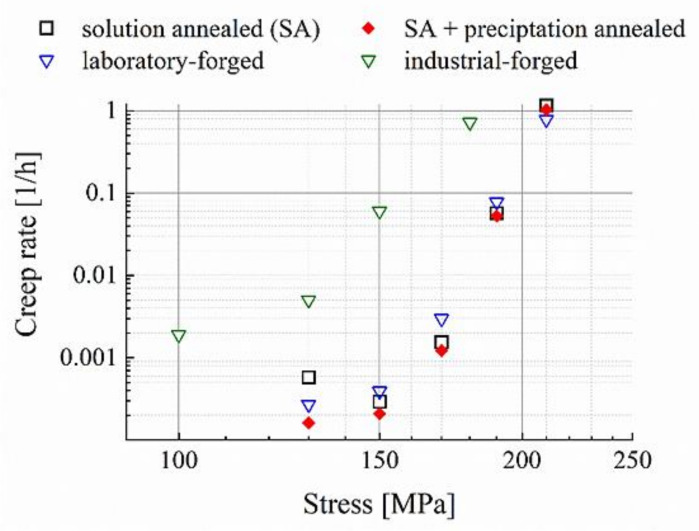
Creep rate over stress relations of the Fe17Cr2.6W1Nb trial alloy in various processing variants (650 °C).

Figure 6 displays the secondary creep rates over the applied creep stress of the alloy in different microstructural states, i.e., processing variants. With the exception of 130 MPa, the creep rates of the solution-annealed material correlates well with the laboratory-forged state. The solution-annealed material enters the stepped-stress creep experiment at 130 MPa without a defined population of strengthening precipitates. This causes the initial comparably-high creep rate, which, because of precipitation, then drops below the values of the laboratory-forged and the solution-annealed plus precipitation treated [8] material at the higher testing stress rates further in the course of the experiment. In other words, the experiment starts with an unstable material microstructure, which increasingly self-stabilizes, because of precipitate formation during the further course of the creep. The coincidence in the results demonstrates that, in the laboratory scale, creep rates comparable to conventionally quality heat-treated materials are obtainable by process integrated thermomechanically induced precipitation. In case of the industrially forged variant, the encountered creep rates range approximately one to two orders of magnitude higher over the entire range of stress due to the coarser precipitates (Figure 2c).

## 4. Conclusions

With creep properties comparable to conventional quality heat-treatments, integrated thermomechanical processing of a HiperFer trial steel was successfully demonstrated at the laboratory scale. However, the transfer of the thermomechanical treatment parameters, from the laboratory to the industrial scale, has not yet fully succeeded. Thermomechanically induced precipitation of the strengthening Laves phase was successfully achieved at both the laboratory and industrial scale, but at the industrial-scale, forging parameters resulted in a comparably coarse, inhomogeneous distribution of Laves phase particles. The obtained mechanical properties are consistent with the respective microstructures. On the industrial scale, large grain size, coarse precipitates at the grain boundaries and within the grain interiors, in combination with wide PFZs, cause the brittle behavior of the model alloy during Charpy impact testing.

An optimization of the forging parameters is necessary to reach a homogeneous distribution of fine precipitates. The encountered large grain size is caused by only focusing on achieving thermomechanically induced precipitation, and can be controlled in regards to the complete process route, by including two-step forging with integrated dynamical recrystallization. The investigated fully ferritic, high-chromium stainless steels are based on pronounced precipitation hardening by intermetallic Laves phase. Therefore, an optimum PFZ width should be determined, balancing its potentially positive and negative effects. Both grain size and PFZ width adjustment are within the focus of the current research.

## Figures and Tables

**Table 1:** Chemical composition in wt. % of the manufactured model alloy Fe17Cr2.6W1Nb.

C	O	Si	Mn	Cr	Nb	W	Fe
0.002	0.2	0.25	0.19	17.1	0.99	2.6	bal.

## Data Availability

The data presented in this study are available on request from the corresponding author.
